# Bioactive Natural Products 2016

**DOI:** 10.1155/2016/9749305

**Published:** 2016-09-29

**Authors:** Yiannis Kourkoutas, Nikos Chorianopoulos, Kimon A. G. Karatzas, Ibrahim M. Banat

**Affiliations:** ^1^Laboratory of Applied Microbiology and Biotechnology, Department of Molecular Biology and Genetics, Democritus University of Thrace, 68100 Alexandroupolis, Greece; ^2^Institute of Technology of Agricultural Products, Hellenic Agricultural Organization-DEMETER, 15310 Athens, Greece; ^3^Department of Food and Nutritional Sciences, University of Reading, Reading RG6 6AD, UK; ^4^School of Biomedical Sciences, Pharmaceutical Science and Practice Research Group, Ulster University, Cromore Road, Coleraine, County Londonderry, UK

Various preservatives are being used to ensure that manufactured foods remain safe and unspoiled. The excessive use of chemical preservatives, many of which are believed to exert potential carcinogenic activities, as well as residual toxicity, has resulted in mistrust among European consumers. Since consumers need to feel reassured that they consume safe foods, an increasing pressure on food manufacturers and authorities is applied with respect to the elimination of harmful chemical preservatives from food preparations, strengthening the research activity towards the discovery of alternative agents [1, 2]. In this context, the use of natural products presents an intriguing case. They exhibit a wide range of biological and pharmacological activities and are considered to have beneficial effects in human nutrition [1, 2]. Currently, natural products are used in food preparations mainly as flavouring agents [3] and by cosmetic and pharmaceutical industries as fragrances and functional additives [4]. These natural substances have been suggested for use in foodstuffs [5] because they were found to display significant antimicrobial properties against bacteria and fungi [1, 2, 6–8].

The main objective of this special issue was to provide a number of documents focused on the facts, applications, and challenges of bioactive natural products and present the methodologies in use to evaluate the effectiveness of such products. Moreover, the challenges that the industry faces with respect to the use of bioactive natural products as antimicrobial agents in terms of safety and microbial growth prevention are discussed. Hence, the antioxidant, antimicrobial, anti-HIV, and cholinesterase inhibitory activities of aqueous and alcoholic extracts from leaves, stems, and flowers of* Euphorbia characias* were evaluated. It was suggested that* Euphorbia characias* extracts represent a good source of natural bioactive compounds which could be useful for pharmaceutical application, as well as in food systems for the prevention of the growth of food-borne bacteria and to extend the shelf-life of processed foods. Furthermore, it was evident from the findings of another study that* Pterospartum tridentatum* and* Mentha pulegium* may constitute an important reservoir of phytochemicals with antiradical activity and antibacterial capacity and thus they might be used in a preventive way or in a combined pharmaceutical and antibiotic therapy against pathogenic bacteria.

Sulfated polysaccharides extracted from five seaweed samples collected or cultivated in Mexico were tested to evaluate their effect on measles virus* in vitro*. The synergistic effect would allow reduction of the treatment dose and toxicity and minimization of the induction of antiviral resistance. Sulfated polysaccharides of the tested seaweed species appeared as promising candidates for the development of natural antiviral agents.

The main types of natural products that have been characterized as splicing inhibitors, the recent advances regarding molecular and cellular effects related to these molecules, and the applications reported so far in cancer therapeutics were also summarized in the current special issue. Likewise, hyperoside, an active compound found in plants of the genera* Hypericum* and* Crataegus*, is reported to exhibit antioxidant, anticancer, and anti-inflammatory activities. The results presented suggested that hyperoside may have potential as a therapeutic agent for the treatment of liver fibrosis.

Polyphenols from diverse sources have shown anti-inflammatory activity [9, 10]. In atherosclerosis, macrophages play important roles including matrix metalloproteinases synthesis involved in degradation of matrix extracellular components affecting the atherosclerotic plaque stability. These data suggested a potential role of polyphenols from Chilean propolis in the control of extracellular matrix degradation in atherosclerotic plaques. Besides, the results of another investigation indicated that Chilean propolis has a dose-dependent effect on the inhibition of genes involved in* Streptococcus mutans* virulence and adherence through the inhibition of glucosyltransferases, showing an anticariogenic potential of polyphenols from propolis beyond* S. mutans* growth inhibition.

*S. mutans*, with the ability of high-rate acid production and strong biofilm formation, is considered the predominant bacterial species in the pathogenesis of human dental caries [11, 12]. In this vein, LongZhang Gargle, completely made from Chinese herbs, was investigated for its effects on acid production and biofilm formation by* S. mutans*. The findings suggested that LongZhang Gargle may be a promising natural anticariogenic agent as it suppressed planktonic growth, acid production, and biofilm formation against* S. mutans*.

Moreover, the production and incorporation of conjugated linoleic acids (CLA) in biomass were reported for the first time.* B. breve* WC 0421 stored CLA in the form of free fatty acids, without changing the composition of the esterified fatty acids, which mainly occurred in the plasmatic membrane. Finally, 19* Streptococcus thermophilus* strains with high exopolysaccharide production abilities were isolated from traditional Chinese fermented dairy products. The exopolysaccharide and viscosity of milk fermented by these 19 isolates were investigated. It was demonstrated that the selected higher exopolysaccharide producing cultures could be used as yogurt starter cultures reducing the amount of added stabilizer, which can compare favorably with the imported commercial one.

*Yiannis Kourkoutas* *Nikos Chorianopoulos* *Kimon A. G. Karatzas* *Ibrahim M. Banat*
